# Cross-species evidence that nicotine widens the attentional window

**DOI:** 10.1007/s00213-021-05972-y

**Published:** 2021-10-07

**Authors:** Britta Hahn

**Affiliations:** 1 Department of Psychiatry, Maryland Psychiatric Research Center, University of Maryland School of Medicine, P.O. Box 21247, Baltimore, MD 21228, USA

**Keywords:** Nicotine, Attention, Spatial attentional resource allocation task, SARAT, 5-choice serial reaction time task, 5-CSRT, Attentional window, Broad monitoring, Non-smokers, Rats

## Abstract

**Rationale:**

The ability to spread attention over items or locations is as important for everyday functioning as the ability to focus narrowly. Little is known about neuronal processes involved in broad monitoring, but indirect evidence suggests a role of nicotinic acetylcholine receptors (nAChRs).

**Objective:**

The present study tested whether the prototypical nAChR agonist nicotine enhances the ability of humans and rodents to maintain a broad attentional window.

**Methods:**

Fifty-three never-smokers wearing a nicotine (7 mg/24 h) or placebo patch performed an attention task requiring detection of stimuli presented randomly in one of four peripheral locations, with a central cue predicting the target location or indicating the need to spread attention over all locations. Nineteen rats performed the 5-choice serial reaction time task requiring detection of stimuli presented randomly in a horizontal array of five locations. Performance after nicotine (0.1 and 0.2 mg/kg) or vehicle administration was analyzed as a function of target location eccentricity.

**Results:**

In human subjects, nicotine caused greater reaction time reduction when all locations were monitored than when a single location was cued. In rats, nicotine attenuated the decline in stimulus detections and the increase in omission errors with greater target location eccentricity.

**Conclusions:**

The findings represent cross-species evidence that nAChR agonism facilitates the ability to spread attention broadly. This suggests that nAChR hypofunction may be central to broad monitoring deficits as seen, for example, in schizophrenia. The homology of findings between the rodent and the human paradigm contributes to validating a translational strategy for treatment development.

## Introduction

Navigating everyday life activities depends on processing the right information at the right time. Selective attention enables us to select parts of the vast array of available sensory input for processing, at the expense of other parts. However, the ability to spread attention over several items or locations is also important for everyday functioning. A broad attentional window enables awareness of our surroundings and the ability to respond to unexpected changes in environmental demands, even when occurring at unpredictable locations. Real-life examples include handling complex traffic situations, supervising a group of small children on a playground, or participating in a basketball match. The detection of critical events in such scenarios depends on expanding the attentional window and spreading the benefits of attentional processing across the visual field (Belopolsky et al. 2007; Eriksen and Yeh 1985).

Over the last decade, cognitive neuroscience studies have produced evidence that people with schizophrenia (PSZ) have broad monitoring deficits and tend to hyperfocus processing resources on a narrow spatial window or a small number of locations or representations (see Luck et al. 2019 for a review of the evidence). One of the first descriptions of broad monitoring deficits in PSZ was based on the spatial attentional resource allocation task (SARAT), in which target stimuli appear randomly in one of four peripheral locations and a central cue predicts the target location with varying precision (Hahn et al. 2006). Compared with predictive and even with invalid cues, target detection in PSZ was most impaired relative to control subjects when the cue was nonpredictive and all four locations had to be monitored (Hahn et al. 2012), suggesting suboptimal maintenance of a broad attentional window.

Little is known about the neuronal processes involved in broad monitoring functions, but indirect evidence suggests a role of nicotinic acetylcholine receptors (nAChRs). The pathophysiology associated with schizophrenia includes nAChR hypofunction (Adams and Stevens 2007; Hong et al. 2011; Petrovsky et al. 2010), which has at times been linked with cognitive deficits in this condition (Young and Geyer 2013). Parallel suggestive evidence came from a rodent paradigm requiring the detection of spatially unpredictable stimuli across a horizontal array of locations, the 5-choice serial reaction time task (5-CSRTT). In this paradigm, broad monitoring deficits in the form of a more pronounced decrement in target detection with greater location eccentricity were found in a rat model of kynurenic acid elevation (Hahn et al. 2018). Kynurenic acid is a non-competitive antagonist of the α7 nAChR subtype (Hilmas et al. 2001), among other effects, and its levels are elevated in the brains of PSZ (Erhardt et al. 2001; Linderholm et al. 2012; Sathyasaikumar et al. 2011). In an early 5-CSRTT study exploring the effects of nicotine on performance by stimulus location, Blondel et al. (2000) found a trend suggesting that the effects of nicotine on response latency may be more pronounced at the outer locations. Furthermore, cholinergic involvement in broad monitoring was suggested by a functional magnetic resonance imaging (fMRI) study reporting that an acetylcholinesterase inhibitor reduced the biasing effect of visuospatial attention on location-specific occipital cortex activation and a behavioral index of spatial attention (Bentley et al. 2004).

The aim of the present study was to evaluate the effects of the prototypical nAChR agonist nicotine on broad monitoring ability in healthy human non-smokers and in intact rats, employing the SARAT and the 5-CSRTT. The analogy described above between broad monitoring deficits in these two paradigms suggests cross-species translatability of findings. Parallel effects of nicotine would help identify the neuropharmacological underpinnings of broad monitoring processes and of deficits therein and may facilitate the development of treatment strategies.

## Methods

### Human subjects

Fifty-three healthy non-smokers (20 male, 28 Caucasian, 21 African American, 2 Hispanic, 2 Asian) contributed data to the present study. Participants were 21–53 years of age (*mean* ± *SD*, 33.5 ±10.5) with 12–22 years of education (15.8 ± 2.3). Twenty-seven of these individuals had participated in a study testing the interaction of nicotine effects with galantamine (Hahn et al. 2020b), and 26 distinct subjects participated in a study testing the interaction of nicotine with propranolol (Hahn et al. 2020a). Both studies employed a 2 × 2 factorial design testing transdermal nicotine and placebo in the presence of p.o. galantamine/propranolol and in the presence of placebo. The present analysis included only the two test sessions in which nicotine and placebo were tested in the presence of p.o. placebo.

Participants were recruited from the local community through Internet advertising, flyers, and referrals and gave written informed consent for a protocol approved by the University of Maryland Baltimore Institutional Review Board. Participants had had no more than 40 cigarettes in their lifetime and no nicotine exposure within the last year. The use of centrally active medications, pregnancy, history of neurological or psychiatric disorders including drug abuse, significant liver or kidney impairment, heart problems, hyper- or hypotension, and learning disability were exclusion criteria for both studies.

### Animals

The experiment reported here was performed in 19 male Wistar rats. These animals had been the control group in a study of the effects of prenatal kynurenine elevation (Hahn et al. 2018).

Sixteen pregnant Wistar dams were obtained from Charles River Laboratories (Frederick, MD, USA). The animals tested here were born at the Maryland Psychiatric Research Center (MPRC). One to four animals were used from each litter. The pups were weaned on postnatal day 21 and grouped-housed. Starting at 6 weeks of age, the animals were housed individually. Housing was in a temperature- and humidity-controlled room, fully accredited by the Association for Assessment and Accreditation of Laboratory Animal Care (AAALAC) and maintained on a 12-h light–dark cycle with lights on at 7 a.m. The animals had free access to water and received a food-restricted diet starting at 8 weeks of age, to maintain them at 85% of their age-appropriate free-feeding body weights. The treatment of animals followed the Principles of Laboratory Animal Care (NIH publication No. 86-23, 1996) and was approved by the Institutional Animal Care and Use Committee of the University of Maryland School of Medicine.

### Experimental paradigms

#### Human

**Fig. 1:**
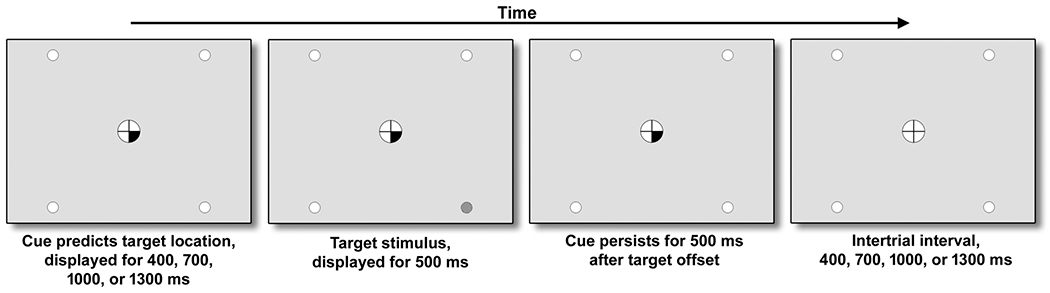
Components of a SARAT trial. Target onset was preceded by a central cue with a variable stimulus-onset asynchrony. Either 1 location was cued as in the figure (predictive cue) or all 4 locations were cued (nonpredictive cue), evoking either a narrow or a broad attentional window in anticipation of the target. Upon detecting a target, participants responded by button press as quickly as possible

The SARAT (Figure 1) was created and run in E-Prime version 2.0 and was performed on a 19-in 5:4 IPS LCD monitor with 1280 × 1024 screen resolution and a 60-Hz refresh rate. Responses were recorded using a Logitech F310 gamepad controller.

The SARAT is a visuospatial stimulus detection paradigm designed to vary the size of the attentional window, from focusing narrowly to monitoring broadly (Hahn et al. 2006). Participants fixate on a quartered circle in the center of the screen, black against a light gray background, and are instructed to respond as quickly as possible when detecting a 500-ms target stimulus appearing in one of four locations in the corners of the screen. The target locations are marked by circular place holders and positioned at ~10° of visual angle. Targets consist of a placeholder circle filling with a gray and white checkerboard pattern of 3 × 3 pixels each. Participants respond with their dominant index finger upon detecting a target. Performance is measured as the reaction time (RT) of target detections and the percentage of omission errors, i.e., trials in which no target response was made.

A cue in the central circle preceded the target signal by 400, 700, 1000, or 1300 ms, chosen randomly. Either one quarter turned black to indicate the location of the upcoming target (predictive cue) or all 4 quarters turned black (nonpredictive cue). Predictive cue trials allowed for a narrow attentional focus, while nonpredictive cue trials required attention to be spread broadly to encompass the entire display. The cue persists for 500 ms after target offset. Only task background is then displayed for a variable intertrial interval (ITI) of 400, 700, 1000, or 1300 ms.

The task was presented in eight 5-min blocks of 60 trials each, including 30 predictive and 30 nonpredictive cue trials. Six predictive and 6 nonpredictive cue trials had no target to discourage anticipatory responding to the cue. To increase the temporal jitter and augment stimulus detection demands, 30 additional 2.7-s periods during which only task background was presented were interspersed randomly between trials. Total task duration was ~45 min.

#### Rat

The 5-CSRTT is a rodent paradigm used extensively to study neuropharmacological and neuroanatomical substrates of attentional processes (Robbins 2002). Operant conditioning chambers (Med Associates, Inc., Fairfax, VT) measuring 26 cm^3^ were housed in sound-insulated enclosures. The curved rear wall contained a horizontal array of five 2.5 cm^2^ apertures, located 2 cm above the grid floor. At the entrance of each hole, a photocell monitored interruptions of an infrared light beam; at the rear, a white light-emitting diode was placed. A food tray was in the opposite wall, equidistant from each aperture. Illumination was provided by a house light in the top portion of the front panel. Apparatus and data collection were controlled by Med-PC software.

All sessions lasted 30 min. Light stimuli (1 s) were presented randomly in one of the five holes after a variable ITI (1–9 s, average 5 s). An equal number of stimuli were presented in each hole over the course of a session. A nose-poke into the hole while it was illuminated or within 5 s after the light had terminated (limited hold) was registered as a correct response and resulted in the delivery of a 45-mg food pellet into the tray, followed by a 2-s reward retrieval period. An incorrect response into any other hole resulted in a 5-s time-out, during which the house light was extinguished. Anticipatory responses during the ITI also resulted in a 5-s timeout. A failure to respond before the end of the limited hold was registered as an omission error.

The following performance measures, collapsed across target locations, are typically reported and are included here for completeness: percentage of correct responses (accuracy), 100 × [correct target detections / (correct + incorrect responses)]; percentage of omission errors, 100 × (omission errors / stimuli presented); latency of correct responses, mean time between stimulus-onset and a nose-poke in the correct hole; and anticipatory responses, total number of ITI responses per session.

To measure broad monitoring ability, performance was parsed as a function of stimulus location as described by Hahn et al. (2018). The following performance indices were derived at each location: (1.) the percentage of all emitted target detections made at this location, 100 × (correct responses at this location / all correct responses emitted), in which this index reflects the relative distribution of target detections in space, independent of the overall number of detections, and (2.) percentage of omission errors, 100 × (omission errors at this location / targets presented at this location). Values reflect the proportion of missed targets at each location. It is important to note that an equal number of stimuli were presented in each hole over the course of a session.

**Fig. 2:**
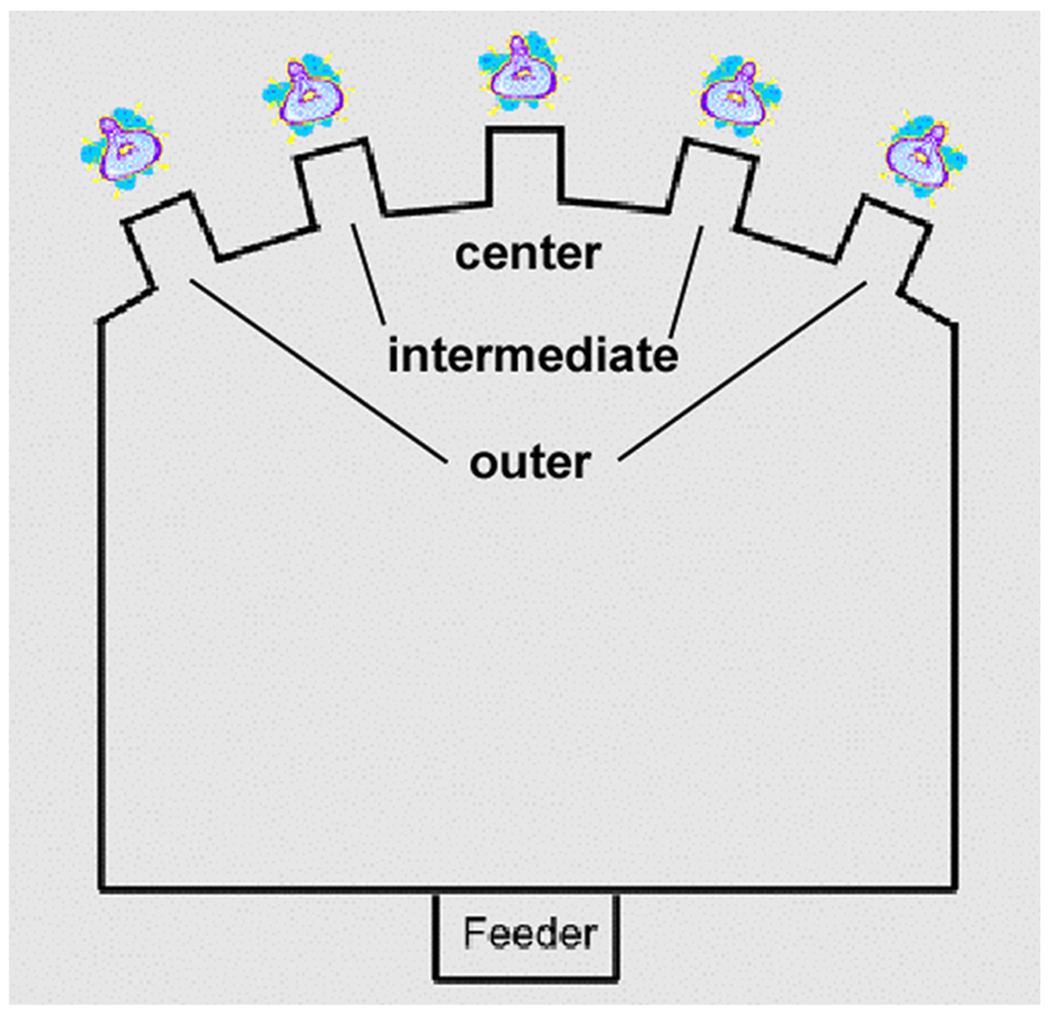
Bird’s eye view of the operant chamber used for the 5-CSRTT. For location-based analysis, the target locations were categorized by eccentricity as outer, intermediate, and center. Performance was averaged over the two intermediate locations and over the two outer locations

Broad monitoring ability is captured by the degree of performance decrement with increasing target location eccentricity: a steeper drop would reflect a narrower attentional window. To this end, the above performance indices were averaged over the two outer locations and over the two intermediate locations (see Figure 2), resulting in three levels of eccentricity. While rats do not necessarily face the center of the array on each trial, a bias to one side would come at the cost of detecting stimuli at the opposite side. Averaging across sides served to cancel out effects of side bias. The motor requirements of reaching each target location are similar due to the curved shape of the wall containing the target locations. Indeed, while performance indices sensitive to stimulus detection (accuracy, omission errors) deteriorate with greater location eccentricity, we find that anticipatory responding in the ITI is unaffected by eccentricity (Hahn et al. 2018).

### Study design and procedures

#### Human

Study procedures were identical between the nicotine–galantamine (Hahn et al. 2020b) and the nicotine–propranolol interaction study (Hahn et al. 2020a), except where stated here. Both studies adopted a double-blind within-subject design in which each participant completed four test sessions, scheduled with at least two intermediate days. On each test day, a skin patch was applied and a capsule administered. On one day, both the patch and the capsule were a placebo (placebo session). On another day, the patch was a nicotine patch and the capsule a placebo (nicotine session). On another day, the patch was a placebo and the capsule contained galantamine or propranolol (depending on the study), and on another day, the patch was a nicotine patch and the capsule contained galantamine or propranolol. These last two conditions were not included in the present analyses. The sequence in which drug conditions were tested was counterbalanced across participants to the degree possible.

Both studies involved six total visits: one consent and screening visit, one training visit, and the four test sessions. During the training visit, participants were given task instructions and performed a full-length version of the cognitive tasks, to minimize practice effects between test sessions. The four test sessions took approximately 7 h each. Upon arrival, participants were tested for fever and for recent drug, alcohol, or tobacco use, all of which had to be negative for the session to proceed. Vital signs were then taken, and participants completed a side effect checklist. Next, the study patch was administered. Vital signs and side effect checks were obtained hourly thereafter. During the drug-absorption period, participants could read, watch movies, or use the Internet. Three or 3.5 h after patch administration, depending on the study, participants swallowed the study capsule, which was always a placebo for the data reported here. Cognitive testing began 5 h after patch application based on reports that nicotine plasma concentrations have reached asymptote by this time and remain stable thereafter, creating an extended testing period despite nicotine’s short half-life (Fant et al. 2000; Gupta et al. 1993). The order of the cognitive tasks was fixed: first the SARAT, then a rapid visual information processing task, and last a change detection task. Only SARAT data are reported here. Cognitive testing was completed within 1.5 h. Immediately after, a 5-ml venous blood sample was obtained for analysis of nicotine concentrations, which averaged 6.2 ng/ml in the nicotine session (Hahn et al. 2020a; Hahn et al. 2020b) — lower than typically found in dependent smokers (Lawson et al. 1998; Russell et al. 1975).

#### Rat

Rats started 5-CSRTT training at 8 weeks of age, following procedures described previously (Mirza and Stolerman 1998). The animals were trained Monday through Friday for 20 weeks. Training commenced with a 30-s stimulus duration. After the basic task contingencies had been acquired, the stimulus duration was progressively shortened to 10, 5, 4, 3, 2, and 1 s. Training and subsequent location-based performance in the 4 weeks preceding the present experiment were reported by Hahn et al. (2018). The experiment presented here began in the week following this 4-week period, 5 weeks after training completion, when rats were 33 weeks of age. Test sessions were conducted twice a week with training sessions on the other weekdays. Test days were always preceded by at least one training day. Task parameters in test sessions were identical to those in training sessions. Nicotine was tested at doses of 0, 0.1, and 0.2 mg/kg, administered subcutaneously (s.c.) 10 min before each test session. No injections were given on training days. The experiment adopted a within-subject design. Thus, each rat was tested three times, once with each dose of nicotine and vehicle, in a sequence that was counterbalanced between animals to the degree possible.

#### Drugs

##### Human

Nicotine patches were over-the-counter Nicoderm CQ patches (GlaxoSmithKline, Brentford, Middlesex, UK) releasing 7 mg of nicotine in 24 h, the lowest dose available. Placebo patches were generated using AquaHeal Hydrogel Bandages (Spenco Medical Corporation), cut to size and with identifying labeling removed. The hydrogel bandages closely resemble the nicotine patch in color and consistency. The nicotine or size-matched placebo patch was placed on the inside of an adhesive bandage on the day of the study and sealed in a small ziplock bag until application. The adhesive bandage with the inserted patch was applied and removed by a nurse not involved in any other study procedures.

##### Rat

(−)-Nicotine tartrate (MP Biomedical, Solon, OH) was dissolved in isotonic saline and the pH adjusted to 7 with NaOH solution. Injections were given s.c. into the flank at a volume of 1 ml/kg. All doses are expressed as those of the base.

### Statistical analysis

#### SARAT

Nicotine effects on mean RT and the percentage of omission errors were analyzed by 3-factor ANOVAs with the between-subject factor of group, coding for which of the two original studies a subject had participated in and within-subject factors of drug (vehicle vs. nicotine) and cue type (1 vs. 4 cued locations). To test whether differences in nicotine effect may be secondary to the overall RT difference between cue conditions, with longer RTs facilitating greater drug-induced reduction, we divided the effect of nicotine (RT under vehicle minus RT under nicotine) within each cue condition by the average RT over drug conditions within this cue condition. The adjusted drug effect was compared between cue conditions by a paired *t* test.

#### 5-CSRTT

For analyses collapsed across locations, response accuracy, the percentage of omission errors, response latency, and anticipatory responses underwent 1-factor ANOVA with drug (0, 0.1, 0.2, mg/kg of nicotine) as within-subject factor. For location-based analyses, the percentage of emitted target detections and the percentage of omission errors made at each location eccentricity were analyzed by 2-factor ANOVA with drug (0, 0.1, 0.2 mg/kg of nicotine) and location (outer, intermediate, center) as within-subject factors.

## Results

### SARAT

**Fig. 3:**
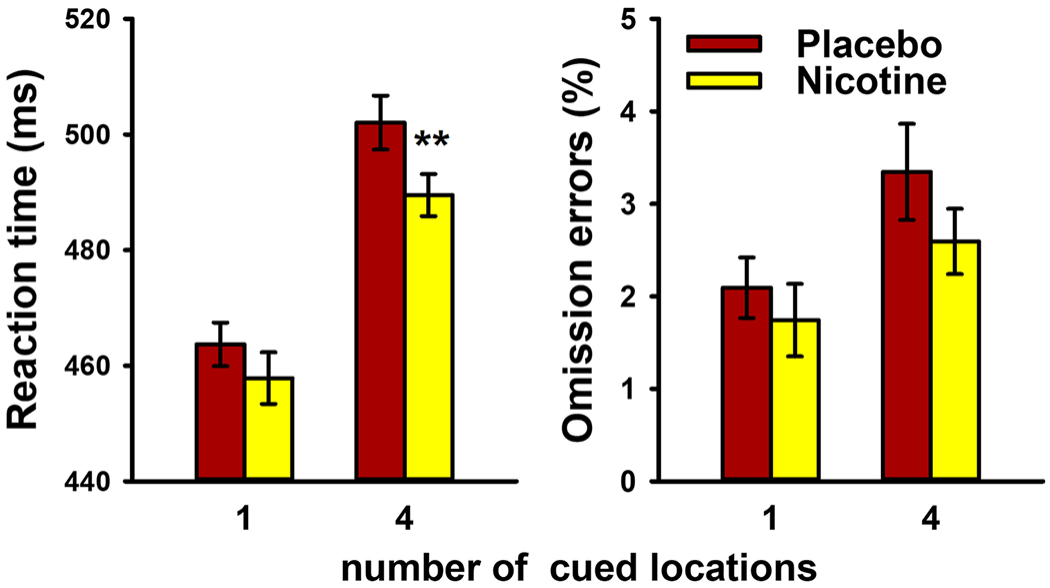
Effects of transdermal nicotine (7 mg/24 h) vs. placebo on average (± SEM) reaction time and omission errors in the SARAT, separately for trials with 1 cued location (predictive cue trials) and 4 cued locations (nonpredictive cue trials). Bars represent the average (± SEM) of 53 non-smokers. **P < 0.01 in paired *t* test

Figure 3 shows effects of nicotine vs. placebo on RT (left panel) and omission errors (right panel). Participants’ responses were slower in nonpredictive than in predictive cue trials [main effect of cue type on RT, *F* (1,51) = 86.3, *P* < 0.001], Nicotine significantly reduced RT [main effect of drug, *F* (1,51) = 6.32, *P* = 0.015], but this effect differed between cue types [interaction, *F* (1,51) = 10.4, *P* = 0.002]. Effects of nicotine were significant in nonpredictive cue trials [*t* (52) = 3.28, *P* = 0.002] but not in predictive cue trials [*t* (52) = 1.53, *P* = 0.13], When adjusting for overall RT in the two cue conditions as described above, the nicotine effect was still significantly larger in nonpredictive than in predictive cue trials [*t* (52) = 2.87, *P* = 0.006]. Neither the group × nicotine interaction [*F*(1,51) = 2.62, *P* = 0.11] nor the group × nicotine × cue type interaction [*F* (51) = 0.04, *P* = 0.84] were significant, indicating that the pattern of effects seen with nicotine did not differ between the two original studies.

Omission errors were rare, averaging 2.4% overall. There were more omission errors in nonpredictive than in predictive cue trials [*F* (1,51) = 6.11, *P* = 0.017]. Neither the main effect of drug [*F* (1,51) = 2.53, *P* = 0.12] nor the drug × cue type interaction [*F* (1,51) = 0.96, *P* = 0.33] were significant were any interactions involving group [group × nicotine, *F* (1,51) = 2.25, *P* = 0.14; group × nicotine × cue type, *F*(51) = 0.01, *P* = 0.91].

### 5-CSRTT

#### Performance collapsed across locations

**Fig. 4:**
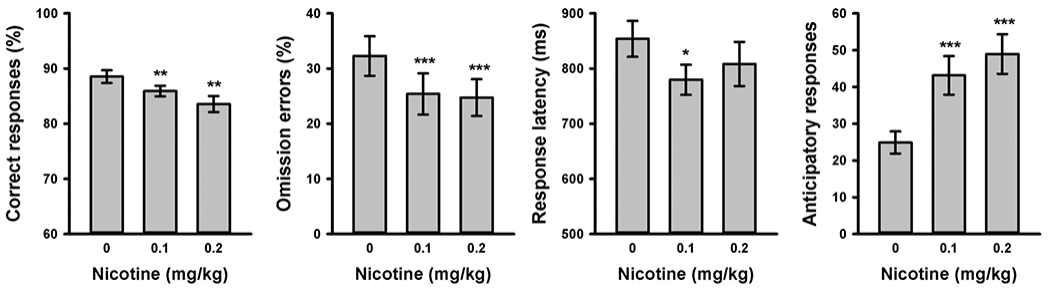
5-CSRTT performance collapsed across locations after systemic administration of vehicle and 0.1 or 0.2 mg/kg of nicotine. Bars represent the average performance (± SEM) of 19 rats in 30-min test sessions. Conditions in which nicotine produced a significant difference compared to saline are marked (*P < 0.05, **P < 0.01; ***P < 0.001, paired *t* test)

As shown in Figure 4, nicotine reduced omission errors [*F* (2,36) = 12.9, *P* < 0.001] and the latency of correct responses [*F* (2,36) = 3.89, *P* = 0.030] and increased anticipatory responding in the ITI [*F* (2,36) = 21.6, *P* < 0.001]. Nicotine did not increase but decreased the percentage of correct responses [*F* (2,36) = 8.49, *P* = 0.001], an effect previously described as secondary to increased anticipatory responding (Hahn et al. 2018; Hahn et al. 2002b). Anticipatory responses have detrimental effects on the accuracy of subsequent target responses because they interfere with scanning potential target locations. Indeed, response accuracy and anticipatory responses were negatively correlated in the vehicle condition [*R* = −0.66, *P* = 0.002]. The location-based analysis avoids this confound by focusing on the spatial distribution of successful target detections.

#### Location-based analysis

**Fig. 5:**
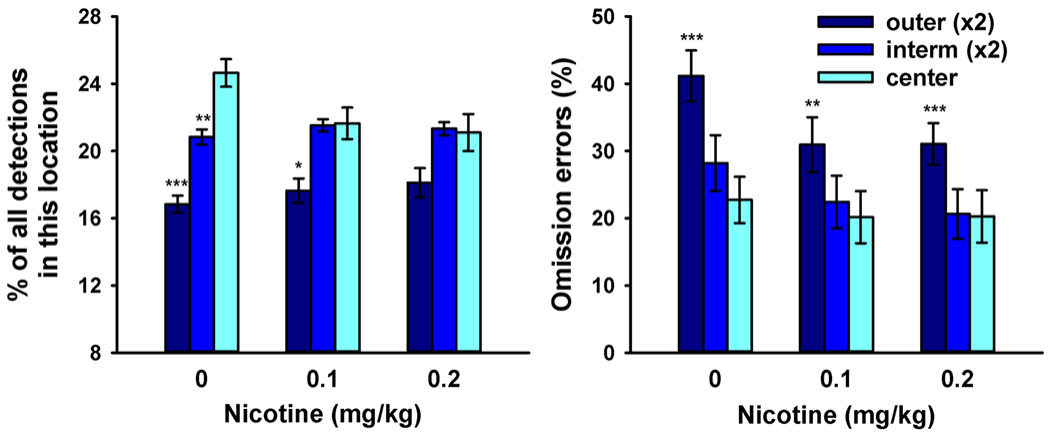
Average (± SEM) 5-CSRTT performance of 19 rats after systemic administration of vehicle and 0.1 or 0.2 mg/kg of nicotine for the outer, intermediate, and center location eccentricities as illustrated in Figure 2. The graph on the left represents the percentage of all correct target responses that were made at each location eccentricity. The graph on the right shows the percentage of omission errors out of all stimuli presented at each location eccentricity. Values were averaged over the two intermediate and the two outer locations. Conditions in which performance at the intermediate or outer location eccentricity was significantly worse than at the center location are marked (*P < 0.05, **P < 0.01; ***P < 0.001, paired *t* test)

Figure 5 (left) shows that a larger proportion of emitted correct responses were made at more central target locations [main effect of location, *F* (2,36) = 12.7, *P* < 0.001]. Nicotine flattened the relative distribution of target detections across location eccentricities, as supported by a significant drug × location interaction [*F* (4,72) = 5.33, *P* < 0.001]. Follow-up 1-factor ANOVAs found a significant effect of location in the presence of vehicle [*F* (2,36) = 28.0, *P* < 0.001] and, reduced, also in the presence of 0.1 mg/kg of nicotine [*F* (2,36) = 7.10, *P* = 0.003], but only a trend was seen in the presence of 0.2 mg/kg of nicotine [*F* (2,36) = 3.20, *P* = 0.053].

Figure 5 (right) shows that more omission errors were being made at more peripheral target locations [main effect of location, *F* (2,36) = 27.1, *P* < 0.001]. This effect was reduced by nicotine, as supported by a significant drug × location interaction [*F* (4,72) = 3.55, *P* = 0.011]. Follow-up 1-factor ANOVAs found a significant effect of target location in each of the three drug conditions (all *P*s < 0.001). Nicotine significantly reduced omission errors at the outer locations [F (2,36) = 14.7, *P* < 0.001] and, to a smaller degree, at the intermediate locations [*F* (2,36) = 6.68, *P* = 0.003] but not at the center location [*F* (2,36) = 1.05, *P* = 0.36].

## Discussion

The present study demonstrated, in both a human and a rodent paradigm of attention, that small doses of nicotine widen the attentional window and facilitate broad monitoring when required. In human participants, this conclusion is based on the finding that nicotine sped up target detection predominantly when a cue had indicated that the target could show at any of the peripheral locations, as opposed to when the cue had prompted a narrow focus on one expected target location. While the corresponding effect on omission errors was not significant, most likely due to floor effects, the interaction on RT by itself is informative. Attention speeds up perceptual information processing and thus shortens the time required to reach detection threshold (Luck and Vecera 2002; Palmer 1998). Thus, faster RT, especially when seen in a task condition-specific manner, is likely to reflect enhanced attention and not psychomotor stimulant effects. In rats, the conclusion that nicotine facilitates broad monitoring is based on measures related to stimulus detection; nicotine evened out the spatial distribution of target detections across eccentricities, and it reduced the percentage of missed targets predominantly at more peripheral locations.

The finding in the 5-CSRTT is consistent with an earlier study in which performance was analyzed across the five target locations (Blondel et al. 2000). While the effects of nicotine in this study were limited to decreasing response latency and increasing anticipatory responding, a trend interaction suggested that the effects on response latency were more pronounced at the more peripheral locations, resulting in a tendency to equalize latencies across locations.

In the present study, the finding that nicotine equalized target detections across location eccentricities is overshadowed by the fact that nicotine decreased response accuracy overall. Increased exploratory behavior in the ITI has been a long-known confound when employing the 5-CSRTT to probe nicotine for attention-enhancing properties (Blondel et al. 1999; Hahn et al. 2018; Hahn et al. 2002b), and the negative relationship between anticipatory responding and response accuracy was confirmed in the present dataset, as well. The tendency to engage with individual unlit target locations is maladaptive for scanning all locations. Even in trials in which this did not result in an anticipatory response and ensuant time-out, this tendency can be expected to interfere with the correct localization of the target stimulus because the rat is more likely to be oriented elsewhere when the stimulus appears. Thus, an increase in this type of behavior would reduce the accuracy of target responses. In addition, in some trials, an anticipatory response may coincide with the onset of a target stimulus and be registered as an incorrect response, thus reducing response accuracy.

Anticipatory responding is a measure of non-specific response rate associated with impulsivity (Bari and Robbins 2013) — a construct separate from attention but which can interfere with the measurement thereof. Psychostimulants like nicotine increase non-specific response rate in animals. Effects on rate-independent measures of response choice, reflecting attention, can be discerned only if interference such as described above can be avoided. In the 5-CSRTT, this had previously been achieved using small doses of nicotine and by not punishing anticipatory responses (Bizarro et al. 2004; Hahn et al. 2002a; b; Hahn et al. 2011; Hahn and Stolerman 2002; 2005; Mirza and Stolerman 1998). The latter resulted in high rates of anticipatory responding overall, but the additional increase induced by nicotine was more subtle, causing less interference with its effects on the accuracy of target responses. In the present study, anticipatory responses were punished by time-outs, creating suboptimal conditions for studying the effects of nicotine on response accuracy. The decision to punish anticipatory responses was made based on consideration in the context of the parent study for which the present animals were the control group (Hahn et al. 2018). Importantly, the location-based analysis of target detections adopted here to study the width of the attentional window avoids the confound posed by non-specific response rate effects because it focuses on the relative spatial distribution of only successful target detections that have been emitted.

The parallel finding in the human paradigm benefitted from the ability to combine two individual studies employing almost identical procedures. Thus, while the interaction reported here had transpired previously (Hahn et al. 2020b), analysis of the combined larger sample yielded the most convincing evidence to date that nicotine helps widen the attentional window, trumping the fact that the critical interaction had not been significant in three other studies employing smaller sample sizes (Hahn et al. 2013; Hahn et al. 2020a; Hahn et al. 2007).

There are some obvious differences between the task demands of the rodent 5-CSRTT and the human SARAT. First and foremost, the 5-CSRTT harbors motor demands that have no equivalent in the SARAT. While human subjects remain stationary and are instructed to not move their eyes from the central fixation cross, rats move around the chamber — to collect food reward, to reorient themselves back toward the target locations, and to approach and respond into the apertures harboring the target stimuli. This difference in motor requirements can explain why the 5-CSRTT, but not the SARAT, is vulnerable to confounds from psychomotor effects of stimulants like nicotine. While not unavoidable, the difference in motor demands represents a species-appropriate behavioral approach to measuring cognitive constructs, and, for the reasons outlined above, the measurement of broad monitoring ability in the 5-CSRTT is unaffected by confounds related to response rate. In both paradigms, the ability to expand the attentional window and monitor broadly is measured in a manner that is unaffected by overall performance, as a performance difference — either between two conditions (SARAT) or between location eccentricities (5-CSRTT). A difference between paradigms is that broad monitoring ability in the SARAT is determined relative to performance when directing attention narrowly, a condition that is not included in the 5-CSRTT. Thus, it may be possible to increase the analogy between paradigms. However, whether this would enhance the inter-species translatability of the construct of interest is unclear. The homology between the rodent and the human paradigm of the present findings with nicotine suggests a useful translational strategy for studies aimed at understanding and treating broad monitoring deficits.

The present findings advance the understanding the neuropharmacological processes involved in maintaining a wide attentional window. This may constitute a first step toward understanding possible mechanisms underlying broad monitoring deficits such as seen in schizophrenia (Luck et al. 2019), as well as in mild cognitive impairment (MCI) or Alzheimer’s disease (AD) where this type of deficit appears to be predict traffic safety (Stern et al. 2016). Indeed, both schizophrenia and MCI/AD have been associated with nAChR hypofunction (Adams and Stevens 2007; Kendziorra et al. 2011), consistent with the present finding that a nAChR agonist facilitates broad monitoring. Replicating broad monitoring deficits with a nAChR antagonist would further support the idea that these deficits are related to nAChR hypofunction. Along the same lines, broad monitoring deficits may contribute to the apparently greater accident proneness among individuals in nicotine withdrawal (Waters et al. 1998). Finally, the present finding that nicotine broadens the attentional window suggests a way of manipulating broad monitoring ability, with possible therapeutic implications.

In summary, the present study provides inter-species evidence that nicotine can help widen the attentional window in a task-adaptive manner. This finding opens the door to investigations aimed at developing interventions for an overly narrow attentional focus, which can pose limitations to everyday life functioning and safety.
